# Autologous transplantation of intestine-isolated glia cells improves neuropathology and restores cognitive deficits in β amyloid-induced neurodegeneration

**DOI:** 10.1038/srep22605

**Published:** 2016-03-04

**Authors:** Giuseppe Esposito, Giovanni Sarnelli, Elena Capoccia, Carla Cirillo, Marcella Pesce, Jie Lu, Gaetano Calì, Rosario Cuomo, Luca Steardo

**Affiliations:** 1 Department of Physiology and Pharmacology, “La Sapienza” University of Rome, Italy; 2 Department of Clinical Medicine and Surgery, University of Naples “Federico II”, Naples, Italy; 3 Laboratory for Enteric NeuroScience (LENS), TARGID, University of Leuven, Leuven, Belgium; 4 Department of Neurology, Beth Israel Deaconess Medical Center, Harvard Medical School, Boston, Massachusetts, USA; 5 Institute of Experimental Endocrinology and Oncology-CNR. Naples, Italy

## Abstract

Alzheimer’s disease (AD) is characterized by chronic deposition of β-amyloid (Aβ) in the brain, progressive neurodegeneration and consequent cognitive and behavioral deficits that typify the disease. Astrocytes are pivotal in this process because they are activated in the attempt to digest Aβ which starts a neuroinflammatory response that further contributes to neurodegeneration. The intestine is a good source of astrocytes-like cells-referred to as enteric glial cells (EGCs). Here we show that the autologous transplantation of EGCs into the brain of Aβ-injected rats arrested the development of the disease after their engraftment. Transplanted EGCs showed anti-amyloidogenic activity, embanked Aβ-induced neuroinflammation and neurodegeneration, and released neutrophic factors. The overall result was the amelioration of the pathological hallmarks and the cognitive and behavioral deficits typical of Aβ-associated disease. Our data indicate that autologous EGCs transplantation may provide an efficient alternative for applications in cell-replacement therapies to treat neurodegeneration in AD.

A chronic, progressive imbalance in either Aβ production or removal rates due to malfunctioning resident astrocytes in the brain plays a crucial role in AD progression^1^,^2^.

In response to Aβ deposition, resident astrocytes are activated, proliferate and migrate to the site of amyloid plaques to digest Aβ^3^,^4^. However, during this process, astrocytes also release proinflammatory factors that intensify the progressive neuronal degeneration and loss in the Alzheimer’s disease (AD) brain^5^,^6^,^7^. So far, few attempts have been made with the possible use of astrocyte-targeted treatments, with noticeable success in animal models of AD^4^,^6^,^7^,^8^. In particular, the idea of replacing malfunctioning astrocytes with brand new ones, proposed by *Pihlaja et al.*^4^, is very challenging and bears a huge therapeutic potential. However, many limitations exist on allogenic transplantation and its potential applications to humans^9^,^10^. A possible strategy could be the use of astrocytes-like cells, namely enteric glial cells (EGCs) in the intestine, as these share many morphological and functional features with cerebral astrocytes^11^. Here we evaluated the efficacy of the autograft of EGCs in the brain of a rat model of Aβ-induced AD (Fig. 1a). We first demonstrated astrocyte activation following the infusion of Aβ_(1–42)_ peptide in rat brains. Specifically, after Aβ-plaque deposit and growth in both the cortex and hippocampus (Fig. 1b) we found that astrocytes (GFAP positive cells) surrounded Aβ deposits, confirming previous evidence^3^. In transplanted rats, where the PKH26GL marker indicates the successfully engrafted cells, EGCs migrate and visibly surround Aβ_(1–42)_ plaques over the time, thereby behaving like resident astrocytes (Fig. 2a,b). In addition, and differently to astrocytes, engrafted EGCs significantly and time-dependently reduced the uptake of ^11^C-labeled Pittsburgh Compound-B ([^11^C]-PIB), as assessed by PET scans using the [^11^C]-PIB radiotracer, which specifically binds fibrillary Aβ plaques^12^ (Fig. 1d,e); accordingly, the size of Aβ burden (Fig. 1c) and plaques size (Fig. 2c,d) were significantly decreased, as shown by the quantitative analysis in brain slices.

To evaluate whether the effect of EGCs was due to their ability to digest Aβ aggregates, we measured *in vitro* the expression of specific proteases, namely neprilysin and Endothelin-converting enzyme-2 (ECE-2), which are known to be involved in amyloid clearance^13^,^14^. The result was a significant upregulation of both proteases when EGCs cultures were exposed to Aβ_(1–42)_ peptide (Supplementary Fig. 1). This result was confirmed *in vivo* in transplanted rats, where EGCs overexpressed neprilysin and ECE, and induced Aβ degradation with a significant peak at eight weeks (Supplementary Fig. 2). As evidence of Aβ_(1–42)_ deposit, we looked at the size and density of neurofibrillary tangles (NFTs) in rat brains. In line with the above results, we found that the engraftment of EGCs caused a marked reduction of NFTs density, with a peak at eight weeks following transplantation (Fig. 3a,b). Together with the migration to the site of Aβ accumulation, to participate to the clearance of the plaques, reactive astrocytes also secrete pro-inflammatory mediators and neurotoxic factors that amplify the neurodegeneration described in AD brain^15^. Based on the evidence that different mediators and cytokines increase in human AD brain and in animal models^16^, we measured the expression of COX-2, Iba-1, GFAP and phosphorylated tau proteins as well as the release of TNF-α, PGE_2_ and IL-6. After the infusion of Aβ_(1–42)_ peptide in rat brains we found that the expression of proinflammatory mediators and cytokines was dramatically increased in the cortex and hippocampi, thus confirming previous data^7^,^15^,^16^. In contrast, after transplanting the EGCs, we measured a significant attenuation of Aβ-induced neuroinflammation, as shown by the reduced expression of COX-2, Iba-1, GFAP and phosphorylated tau protein, along with a significant decrease in TNF-α, PGE_2_ and IL-6 (Fig. 3c–f). In addition to pro-inflammatory cytokines, many other proteins are released into the brain interstitial space, possibly accounting for the neurotoxicity during AD progression^17^. Aquaporin 4 (AQP-4) has been identified as having a role in the extracellular elimination of ‘waste’ products in the brain, such as Aβ aggregates^18^. AQP-4 is expressed on the endfeet of astrocytes and is part of the so called ‘glymphatic unit’^19^. Thus, we wondered whether AQP-4 was involved in EGCs-mediated Aβ clearance. We show here that the expression of AQP-4 was increased at the brain-liquor interface after EGCs transplantation (Supplementary Fig. 3a–c). Although more sophisticated approaches are needed to demonstrate that EGCs-associated AQP-4 upregulation participates to the elimination of proteases-degraded amyloid plaques, our data showing that in EGC-transplanted rats there is a significant reduction of tissue Aβ and an increased excretion in cererebrospinal fluid (Supplementary Fig. 3d,e) likely support what previously hypothesized by Weller and colleagues^20^.

There is evidence that rat primary EGCs are able to release neurotrophines *in vitro*, indicating that these cells have the potential to promote neurogenesis, or at least, to have neurotrophic effects^21^. Here we confirm these data, showing that EGCs maintain this ability also *in vivo.* Starting at two weeks post-transplantation and reaching a peak at eight weeks, the concentration of NGF, BDNF and GDNF was significantly higher in EGCs-transplanted rats (Fig. 4a,c), with a close contact between transplanted EGCs and neurotrophines-expressing fibers being evident at eight weeks (Fig. 4b). Interestingly, engrafted EGCs appear to promote neurogenesis specifically in the areas of Aβ_(1–42)_-injection. We indeed found a significantly increased expression of Doublecortin X, a marker of newborn neurons, at eight weeks after EGCs transplantation (Fig. 4d,e); at the same time point, we also found a significantly increased number of BrdU-labeled cells (Fig. 4f,g), which supports the hypothesis that EGCs may affect, at least partially, the neurogenesis.

In recent years, stem cell transplantation has been proposed for the treatment of neurodegenerative disorders^22^. Generating human pluripotent stem cells raises ethical and safety concerns, which strongly limit their therapeutic potential in humans. Cultured EGCs may theoretically contain neuronal progenitors that could have stem cell-like profile. To check whether the results we observed were due to adult EGCs or to the presence of neuronal or glial progenitors, we stained EGC cultures with SOX-2 and CD133, which are markers of stem cells^23^,^24^. We found that EGCs did not express either SOX-2 or CD133, while they did express S100B and vimentin as markers of adult EGCs (Supplementary Fig. 4).

Aβ-induced neurodegeneration is known to induce cognitive and behavioral deficits^25^,^26^. To test whether EGCs transplantation could improve cognitive and behavioral deficits we performed a Morris Water Maze test to measure learning skills and spatial memory retention in rats^27^. During the retention test, control animals spent a significantly longer time in the quadrant than Aβ_(1–42)_-infused rats. Interestingly, after EGC engraftment a significant increase in the time spent on, and the number of crossings over the platform position was also observed (Fig. 5a,b). Similar results have been reported for the object recognition test, which is used to assess recognition memory in experimental models of neurodegenerative diseases^28^. During the trial test, only an impairment in the recognition of the object was observed in Aβ_(1–42)_-injected rats, while after EGC transplantation a significant amelioration of the performance was measured (Fig. 5c).

In conclusion, this study provides the first evidence on how EGCs, likely because of their similarity with brain astrocytes, may represent a valid source that, when transplanted into the brain of rats with AD-like neuropathology can reach damaged areas and promote structural and functional neuronal recovery, likely by secreting neurotrophines in their surrounding environment in concentrations that can prompt neurogenesis. Although further studies are needed to better characterize EGCs in the light of the new acquisitions regarding their heterogeneity in different animals^29^,^30^, many considerations further support the possible translation of our results into humans. First, the isolation of mature EGCs is safe and easily performed, as it does not require any invasive neurosurgical procedure. As brain biopsy in humans has many risks and limitations, the appendicectomy is a quite safe procedure and would allow to harvest adult EGCs from the nervous system in the gut (the ENS), as previously described^31^. Second, transplanted EGCs were autologous with a virtually absent risk of immune-mediated graft rejection. Third, transplantation of EGCs into the brain resulted in a substantial improvement of Aβ burden and of the associated neurocognitive decline at a two-month follow-up.

In this perspective, this study lays the foundation for the use of autologously transplanted EGCs in humans and paves the way for new opportunities for the clinical use of astrocyte-like cells based therapies to treat AD and other incurable neurodegenerative disorders in humans.

## Methods

### Cell culture medium, chemicals and antibodies

Dulbecco’s Modified Eagle’s Medium (DMEM)-F12, sterile PBS 1X, trypsin EDTA, antibiotic/antimycotic mixture, protease, collagenase, PKH26GL tracker dye, as well as other chemicals were purchased by Sigma (Sigma-Aldrich, Milan, Italy); Aβ_(1–42)_ peptide was from Tocris (Bristol, UK); cell culture plates and other plastic-consumable materials were from Corning (Lowell, MA, USA). Congo-red, paraformaldehyde and other reagents for immunohistochemistry and histology were from Sigma; mounting medium for immunohistochemistry and immunofluorescence samples Eukitt was from Bioptica (Milan, Italy). Milk blocking buffer was from Sigma (Sigma-Aldrich, Milan, Italy). The following antibodies: mouse anti-S100B, rabbit anti-SOX-2, rabbit anti-vimentin, rabbit anti-CD133, anti-COX-2, rabbit polyclonal anti-GFAP, rabbit polyclonal anti-nerve growth factor (anti-NGF) rabbit polyclonal anti-Glial Derived Neurotrophic Factor (anti-GDNF), rabbit polyclonal anti-Brain Derived Neurotrophic Factor (anti-BDNF), rabbit polyclonal Anti Doublecortin X (anti-DCX); rabbit polyclonal anti-aquaporin-4 antibody (anti AQP-4), rabbit monoclonal anti-NeuN (anti-NeuN), rabbit polyclonal anti-Bromodeoxyuridine (anti-BrdU) and rabbit polyclonal anti β-actin were all from Abcam (Abcam, Cambridge, UK). Rabbit polyclonal Anti phosphor-tau (pSer202) was from Sigma, (Sigma-Aldrich, Milan, Italy); anti-Iba-1 was from Santa Cruz (Santa Cruz Biotech. Inc., Costa Mesa, CA, USA); rabbit anti-endothelin-2 converting enzyme (anti-ECE-2) was from Bioss Inc, (Woburn, MA, USA) and rabbit anti anti-neprilysin was purchased from Novus Biological Europe (Cambridge, UK). Secondary FITC or TRITC-conjugated species-specific antibodies and DAPI were from SIGMA (Sigma-Aldrich, Milan, Italy). Specific horseradish peroxidase (HRP)-conjugated secondary antibodies for Western blot were from Dako (Dako, Golstrup, DK). Other reagents for Western blot detection and analysis were from Amersham (Milan, Italy) and Biorad Laboratories (CA, USA). Amyloid Plaque Stain Reagent Amylo-Glo^®^ RTD™ was from Biosensis (Thebarton, Australia). Enzyme-linked immunosorbent assay (ELISA) kits for the quantification of tumor necrosis factor-α and interleukin-6 (IL-6), Beta amyloid 1–42 (Aβ-42) were from Life technologies (Invitrogen, Life tech. Monza, Italy); PGE_2_ ELISA kit was from Abcam (Cambridge, UK); ELISA kit for GDNF and NGF were from Biosensis (Thebarton, Australia); BDNF ELISA kit was obtained from Mybiosource (San Diego, CA, USA).

### Animals and surgical procedures

Adult male Sprague-Dawley rats (200–250 g) were from Charles River (Calco, Italy). Animals were housed in a pathogen-free barrier facility under a 12-h light/dark cycle, with *ad libitum* access to food and water. Animals were randomly divided into two groups receiving either toxic Aβ 1–42 peptide (*n = *120) or its vehicle infusion (*n = *60) represented by artificial sterile cerebrospinal fluid (CSF) in a solution consisting of (in mM): 119 NaCl, 26.2 NaHCO3, 2.5 KCl, 1 NaH_2_PO4, 1.3 MgCl_2_, 10 glucose. Aβ infusion was performed through a cannula connected to a mini-osmotic pump device (Alzet 2002, Alza, Palo Alto, CA, USA) and implanted in the right cerebral ventricle according to Paxinos and Watson atlas coordinates (AP-0.3 mm, L 1.2 mm, V 4.0 mm)^32^. Aβ 1–42 was continuously infused into the lateral ventricle at a rate of 300 pmol^12 μl−1^^/^^day−1^ in the 20 days following its implantation, and left in place for further transplantation (see below). Under anesthesia, after stereotaxic surgery, Aβ-infused animals underwent appendectomy according to Delibegovic *et al.*^33^. Rats were gently removed from stereotaxic Kopf apparatus, secured with an adhesive band and placed on the surgical table in the supine position. The abdomen of the animal was shaved and disinfected with povidone-iodine solution and dried with gauze. Laparotomy was performed using a median incision. The caecum-appendix was identified and dissected and rapidly placed in sterile Krebs. Rat abdomen was then closed with sutures; recovery was quick and optimal and no antibiotic treatment was used before, during, or after the experiment. During the entire observational period, all animals were checked for local and systemic complications following the double-surgery. All procedures were approved by La Sapienza University Ethics Committee. Animal care was in compliance with the IASP and European Community (EC L358/1 18/12/86) guidelines on the use and protection of animals in experimental research.

### Isolation and culturing of EGCs from appendices

According to the procedure described by Cirillo *et al.*^31^, after surgical removal, appendices were placed in a cold oxygenated sterile Krebs solution containing (in mM): 117 NaCl, 4.7 KCl, 1.2 MgCl_2_ 6 H_2_O, 1.2 NaH_2_PO_4_, 25 NaHCO_3_, 2.5 CaCl_2_ 2 H_2_O, and 11 glucose under carbogen (5% CO_2_, 95% O_2_) atmosphere equilibrated at pH 7.4. Appendices were then longitudinally cut along the mesenteric border and pinned flat, mucosa up, inside a dissection dish containing an ice-cold Krebs solution that was replaced every 5 min. The tissue was dissected by carefully removing the mucosa and the circular muscle to expose the myenteric plexus. After removal, the myenteric plexus was enzymatically digested for 30 min at 37 °C in a solution containing protease (1 mg/ml) and collagenase (1.25 mg/ml). After this phase, the enteric ganglia were picked up and plated onto coverslips in 6-well culture plates. An amount of Dulbecco’s Modified Eagle Medium (DMEM) F-12, supplemented with 10% heat-inactivated calf serum (FCS) and 1% antibiotic-amtimycotic mixture was added to the cells. After 2.5 weeks, EGC cultures were purified using Dynal-Magnet (Miltenyi Biotec GmbH, Bergisch Gladbach, Germany), according to the manufacturer’s instructions and to the method previously described^31^. The separation step with dynabeads was performed twice in order to eliminate residual fibroblasts/smooth muscle cells within the culture. The resulting EGC enriched cultures (∼1000 × 10^4^ cells/ml) were characterized by immunofluorescence.

### Characterization of EGCs cultures

Isolated and cultured EGCs were washed with 1 × phosphate buffer saline (PBS) and fixed with 4% paraformaldehyde in 1 × PBS. EGCs were blocked in 10% albumin bovine serum 0.1% Triton-PBS solution (blocking buffer) for 90 minutes at room temperature; thereafter they were incubated for 1 hour at room temperature with the following antibodies diluted in 10% w/v milk blocking buffer: mouse anti-S100B (1:500), rabbit anti-SOX-2 (1:400), rabbit anti-vimentin (1:300), rabbit anti CD133 (1:300). Finally, EGCs were incubated for 1 hour at room temperature with the appropriate fluorescent secondary antibodies. Nuclei were stained with DAPI (1:5,000, Sigma, Milan, Italy). Pictures were taken using a camera (Nikon Digital Sight DS-U1) connected to a microscope (Nikon Eclipse 80i; Nikon Instruments Europe) provided with the proper fluorescence filters. Slides were analyzed with a microscope (Nikon Eclipse 80i), and images were captured at 10× and 20× magnification with a high-resolution digital camera (Nikon Digital Sight DS-U1). In some experiments, analysis of immunopositive cells was performed using a specific digital system (NIS-Element Basic Research version 2.30 software).

### EGCs tracking with viable PKH26GL dye

In order to mark and localize transplanted EGCs into the receiving brains with a nontoxic dye, EGCs were labeled with the rhodamine-labeled fluorescent dye PKH26GL Red Fluorescent Cell Linker Kit for General Cell Membrane Labeling following the manufacturer’s instructions. PKH26GL is a red fluorochrome with detection systems in immunofluorescence analysis; its capability to track cells before transplantation has been tested in other studies with stem cells and its stability is favorable for long term *in vivo* studies. Confluent EGCs were detached by trypsin: EDTA (1:25), counted and plated in 6-multiwell plates at a density of 5 × 10^4^ cells/ml and allowed to adhere for 24 h. For autologous transplantation, each single EGCs strain isolated and cultured was collected and strictly marked and catalogued before being tracked and re-injected in the same animal from which it was originated. For PKH26GL tracking, after trypsinization, EGC were washed with DMEM/F12 medium and incubated for 3 min with a 1:250 PKH26GL dye. After this procedure, cells were centrifuged again, the excess of fluorescent dye was removed and, immediately before transplantation, the pellet was re-suspended in artificial CSF.

### Transplantation of EGCs in rat brains

As above described, during the 20 days of infusion, we were able to collect for each single appendicectomized rat a significant amount of auto-transplantable EGCs. After preliminary purification and characterization of EGCs, animals were ready for autologous transplantation. Before this, Aβ infusion was stopped and infused rats (*n = *60) were divided in two experimental groups, depending on whether or not they received autologous EGCs transplantation. For transplantation, 5 × 10^5^^/^^μl^ EGCs were tracked with PKH26GL, suspended in a volume of 10μl CSF and infused for 24 h through the same cannula left in place with Alzet osmotic pump (as above described) these animals were thus conventionally considered as “*transplanted*” group. An equal volume of CSF was infused in the remaining rats (*n = *60) without cell infusion, and this group was thus considered as “*sham*” group, as internal control. Both sham and transplanted groups were thus matched with the third rat group, which was initially exposed to vehicle infusion, and considered as “*vehicle-treated*” group. To minimize the discomfort and/or risk of infection after transplantation, at the end of infusion the cannula was removed.

### Morris water maze test

Animals were trained in a pool filled with water kept at 25 ± 1 °C to avoid hypothermia. White-colored water was poured into a circular pool (diameter, 120 cm; height, 40 cm), and a white platform (10 × 6.5 cm, 21.5 cm high) was placed 1.0 cm below the water level in the middle of a fixed quadrant. According to the experimental design, vehicle, sham, and EGC-transplanted (*n = *10 each group, for each time course interval) rats underwent behavioral tasks at different time points after the transplantation. First, acquisition trials were performed four times daily for 7 days to reach a steady state of escape latency. The rats were allowed to swim freely for 120 s and were left for an additional 30 s on the platform. The intertribal interval during four trials was 75 min. Start positions set at each limit between quadrants were randomly selected for each animal. Rats failing to find the platform were placed on the platform manually. Memory-retention tests were performed at the seventh day after the last training session. The platform was removed and each rat was allowed a free 120 s swim. The number of crossings over a point where the platform had been was counted by replay using a video camera set above the center of the pool and connected to a video tracking system (Ethovision 3.0; Noldus Information Technology BV, Wageningen, Netherlands).

### Object recognition test

Object recognition test was carried out in an open-field arena constructed of black Plexiglas (50 cm × 40 cm × 63 cm). Depending upon the experimental design, (*n* = 10 each group, for each time course interval) rats deriving from the vehicle, sham or EGC-transplanted group at different time course post-transplantation intervals underwent an 8-minutes acquisition trial, during which the animal was placed in the open field in the presence of two identical objects identified as A, represented by a cube or ball and located at 15 cm from the arena wall (acquisition task). Upon completion of the exploration time, animals were returned to their respective cages for 3 hours. After the retention interval, rats were placed back into the box and exposed to the known object A and to a novel object B for additional 8 minutes (test task). The objects were placed in the same locations as the previous ones. The position of the novel object was randomly chosen to avoid preferences not based on novelty. Exploratory behavior was considered with the animal directing its nose toward the object closely (<2 cm), and the amount of time spent exploring each of the two objects was thus recorded. To measure the recognition memory, a recognition index was calculated as the amount of time exploring the familiar object (TA) or the novel object (TB) divided by the total time spent exploring both objects and multiplied by 100, according the following formula: [TA or TB/(TA + TB)] × 100. In the acquisition and retention trials, if the exploration time was <30 s and <15 seconds, respectively, the rats were excluded from the trial.

### Pittsburg compound positron emission tomography (PET) scan

At the end of the behavioral test, in the different experimental groups included in the time course, PET scan was performed on rats (*n = *6) after behavioral studies at time points 2-4-8 weeks post EGC-transplantation. After initial anesthesia, (5% isoflurane in O_2_/N_2_O (3:7)) rats were transferred into a sound-attenuated chamber surrounded by a Faraday cage. Inhalation anesthesia was sustained during the whole procedure (2% isoflurane) and body temperature was maintained at 37 °C. Rats were tail-injected with 1.5 mCi Pittsburg compound B [^11^C]-PIB. Micro-PET imaging was performed using a micro-PET R4 rodent model scanner (CTI Concorde Microsystems, Knoxville, TN, USA). Images were reconstructed using a maximum posteriori probability algorithm. Corrections for dead time, random scattering and attenuation were made for each scan. The drawing region of interest analytical technique was used to obtain the averaged pixel counts of each PET image. According to Thie^34^ the standardized uptake value (SUV) is the relative measure of FDG uptake. The basic expression for SUV is: SUV = *r*(*a*′/*w*) where *r* is the radioactivity concentration [kBq/^ml^] measured by the PET scanner within a region of interest (ROI), *a*′ is the decay-corrected amount of injected radiolabeled FDG [kBq], and *w* is the weight of the subject (rat) [g], which is used a surrogate for a distribution tracer volume.

### CSF sampling for Aβ quantification

Immediately following PET procedure, under deep anesthesia, before euthanasia, percutaneous CSF collection was obtained by stereotaxic-driven puncture in the cisterna magna of the rats, accordingly to procedure described by Mahat *et al.*^35^ A mean amount of 100 μl of CSF was collected from each procedure.

### Post-mortem brain characterization

After behavioral testing and PET scan and CSF sampling, animals were sacrificed by overdosing with pentobarbital and perfused intra-cardially with 200 ml of 0.1% NaNO_2_ in phosphate buffer. For histological analysis (*n* = 10 animals from each group), brains were fixed in 4% paraformaldehyde (pH 7.4) for 24 h and subsequently incubated for 24 h in 30% sucrose for cryoprotection. Coronal sections of the brain measuring 30 μm in thickness were obtained with a cryosectioning microtome and stored at 4 °C in PBS until staining. A series of five equally spaced tissue sections spanning the entire hippocampus and frontal cortex areas were then selected for histological and immunohistochemical analysis for neurofibrillary tangles (NFTs) formation. For biochemical analyses (western blot, ELISA) (*n* = 10 animals from each group) brains were sampled, snap frozen in liquid nitrogen and stored at −80 °C until processing.

### Aβ plaques detection and NFTs staining in brain slices

Aβ plaques were identified by staining with Congo red^36^. Hippocampal and frontal cortex sections were treated with sodium chloride solution (sodium chloride-saturated 80% alcohol containing 0.01% sodium hydroxide) for 20 min at room temperature, then stained with Congo red saturated sodium chloride solution for 1 h and finally dehydrated in absolute alcohol. Free-floating immunohistochemistry for NFTs detection was performed to stain phospho-tau (pSer_202_). Hippocampal and frontal cortex sections were incubated overnight with primary antibodies, then exposed to biotinylated secondary antibodies and finally incubated in horseradish peroxidase complex followed by diaminobenzidine (DAB). Sections to be compared were processed simultaneously under the same conditions to reduce variability during processing^37^.

### Immunofluorescence of brain slices

Immunofluorescence was performed on frontal cortex and hippocampal coronal sections with adjacent slices derived from the different experimental groups. Samples were blocked in 10% albumin bovine serum 0.1% Triton-PBS solution for 15 min and then incubated for 1 h with different antibodies depending from the experimental plan. Both coronal and hippocampal sections were incubated with the appropriate antibodies: rabbit anti-GFAP (1:500), rabbit anti-NGF, (1:200) rabbit anti-GDNF (1:250); rabbit anti-BDNF (1:250), rabbit anti-DCX (1:300); rabbit anti-NeuN (1:300); rabbit anti-BrdU (1:200); rabbit anti-aquaporin-4 (anti-AQP-4 1:250), rabbit anti-ECE-2 (1:250), rabbit anti-neprilysin (1:250). Transplanted EGCs were detected in red at specific rhodamine emission wavelength typical for PKH26GL tracker, according the manufacturer’s instructions. Slices were then counter-stained with Amylo-Glo^®^ RTD™ to visualize fibrillar Aβ plaques according the manufacturer’s instructions. Species-appropriate FITC or TRITC conjugated antibodies (1:250) were applied for 2 h at room temperature, followed by washes in PBS and mounting with PBS/Glycerol 1:1.

### Western blot analysis

The expression of cyclooxygenase-2 (COX-2), ionized calcium-binding adapter molecule 1 (Iba-1), GFAP, and phosphor-tau protein was evaluated in homogenates obtained from both hippocampi and frontal cortices isolated post-mortem in the different animal groups at different time point intervals. The cytosolic fraction was used for the analysis. Protein concentration was determined with Bio-Rad assay kit and equivalent amounts (50 μg) of each sample were separated under reducing conditions in 12% SDS-polyacrylamide minigel. The proteins were transferred onto nitrocellulose membrane according to the manufacturer’s instructions (Bio-Rad Laboratories, Hercules, CA, USA). Depending upon the experiments, the membranes were incubated for 1 h at room temperature with specific anti-COX-2 (1:1000); Iba-1 (1:1200); anti-GFAP (1:1000), phospho-tau_602_ (1:600), anti-βactin (1:100) primary antibodies followed by incubation with specific horseradish peroxidase (HRP)-conjugate secondary antibodies. The immune complexes were developed using enhanced chemiluminescence detection reagents and the protein bands were densitometrically analyzed for relative quantification with a GS-700 imaging densitometer.

### Enzyme Linked Immunosorbent Assay (ELISA)

Enzyme Linked-Immuno-Sorbent Assay (ELISA) assay was carried out in both hippocampus and frontal cortex homogenates isolated post-mortem in the different groups at different time point intervals. According to each respective manufacturer’s instructions, ELISA assay for the quantification of TNFα, IL-6, PGE_2_, GDNF, BDNF and NGF was performed. In other experiments, ELISA for Aβ quantification in the cerebrospinal fluid (CSF) and in whole brain homogenates was performed according to manufacturer’s instructions.

### Statistical analyses

All values are expressed as the mean ± SEM. Statistical differences were determined to be significant at P < 0.05. The specific tests used are described in the figure legends. All analyses were performed using GraphPad Prism software (GraphPad Software, Inc., CA USA). The investigators were not blinded to allocation during experiments and outcome assessment.

## Additional Information

**How to cite this article**: Esposito, G. *et al.* Autologous transplantation of intestine-isolated glia cells improves neuropathology and restores cognitivedeficits in β amyloid-induced neurodegeneration. *Sci. Rep.* **6**, 22605; doi: 10.1038/srep22605 (2016).

## Supplementary Material

Supplementary Information

## Figures and Tables

**Figure 1:**
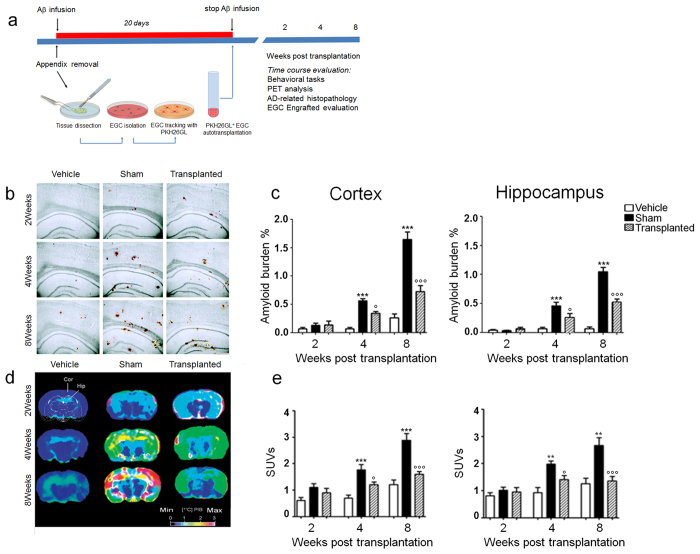
Autologous EGCs induced Aβ plaques degradation. **(a)** Diagram showing Aβ_(1–42)_ peptide infusion, EGCs isolation from the appendix, their transplantation in rat brain, and the time schedule for *in vitro* and *in vivo* measurements. **(b)** The injection of Aβ_(1–42)_ peptide causes a time-dependent amyloid deposition in frontal cortex and hippocampus of treated rats; in EGCs transplanted rats a time-dependent reduction of Aβ plaques was instead observed (*n = *10 per group; congo red staining, scale bar: 500 μm). **(c)** The graphs show that Aβ burden time-dependently increased in sham versus vehicle-treated rats and that EGC transplantation resulted in a significant inhibition of Aβ accumulation. In the frontal cortex (*left panel*) and the hippocampus (*right panel*) Aβ burden was significantly higher in sham (black bars) than in vehicle-treated rats (white bars) at 4 and 8 weeks (***P < 0.001), while in EGC-transplanted rats it was significantly reduced (°P < 0.05 and °°°P < 0.001 vs. sham group). **(d)** Representative scans of ^11^C-labeled Pittsburgh Compound-B positron emission tomography; Standardized uptake values (SUV) images of Aβ binding within the region of interest (ROIs) in the frontal cortex (COR) and hippocampus (HIP) of rats (*n* = 6 per group). **(e)** Relative SUVs for selected ROIs in the cortex (*left panel*) and hippocampus (*right panel*). Data were expressed as mean ± SEM; **P < 0.01, ***P < 0.001 vs. vehicle; °*P* < 0.05; °°° P < 0.001 vs. sham; two-way ANOVA.

**Figure 2:**
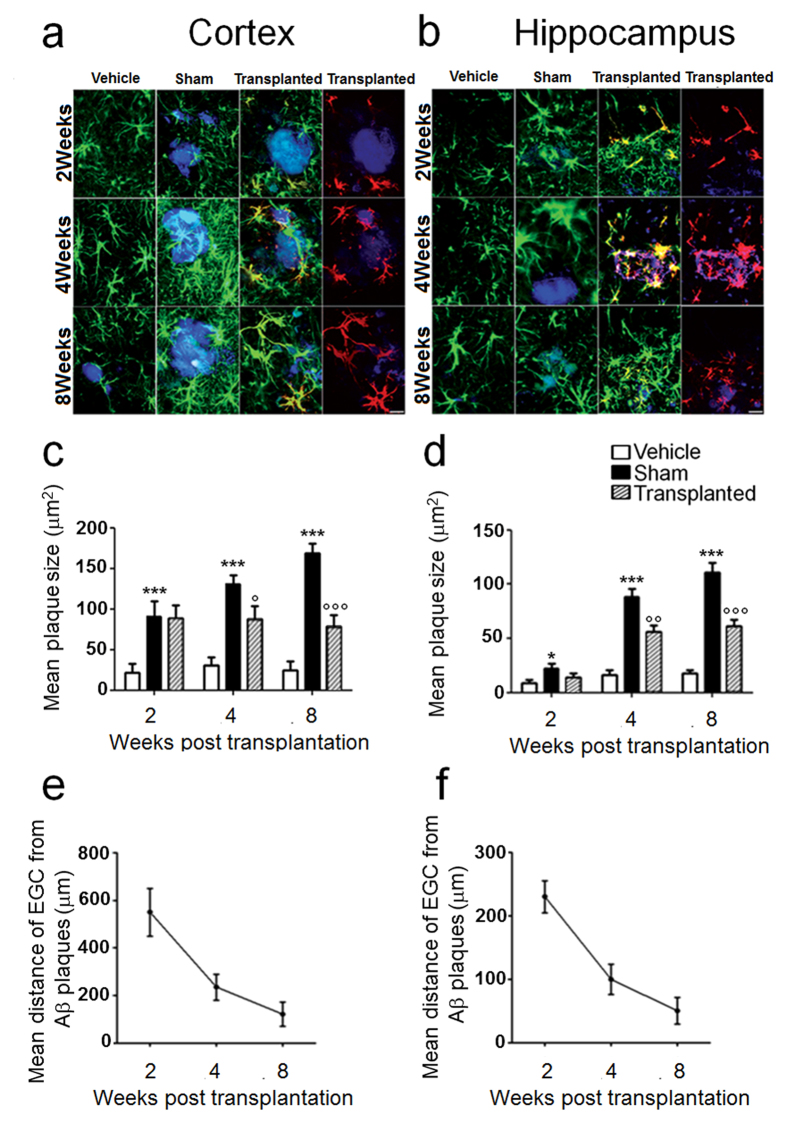
Transplanted EGCs migrate to the site of Aβ plaques deposits. **(a,b)** Immunofluorescence analysis shows that the injection of Aβ_(1–42)_ peptide causes the accumulation of resident astrocytes (GFAP, *green*) around Aβ plaques (*blue*) in the frontal cortex (**a**) and the hippocampus (**b**) of sham compared to vehicle-treated rats. In transplanted rats, EGCs (PKH26GL, *red*) time-dependently migrate to the sites of Aβ plaque deposits in both brain areas and degrade Aβ aggregates at weeks 4 and 8 after injection (*n* = 10 for each group; scale bar: 20 μm). **(c,d)** Plaques size was significantly higher in sham than in vehicle-treated rats at all time points (*P < 0.05 and ***P < 0.001), but in EGC-transplanted rats it was significantly reduced in the cortex (**c**) and the hippocampus (**d**) at 4 weeks and 8 weeks post transplantation (°P < 0.05, °°P < 0.01 and °°°P < 0.001 vs. sham group, respectively). Results were expressed as mean ± SEM of *n* = 6 experiments in triplicate per group. Statistical analysis was performed using parametric one-way ANOVA and Bonferroni’s post-test; *P* < 0.05 was considered significant. **(e,f)** Engrafted EGC time-dependently migrate to the sites of Aβ plaque deposits in both in the frontal cortex (**e**) and the hippocampus (**f**), as revealed by progressive reduction of mean distance from the Aβ deposits.

**Figure 3:**
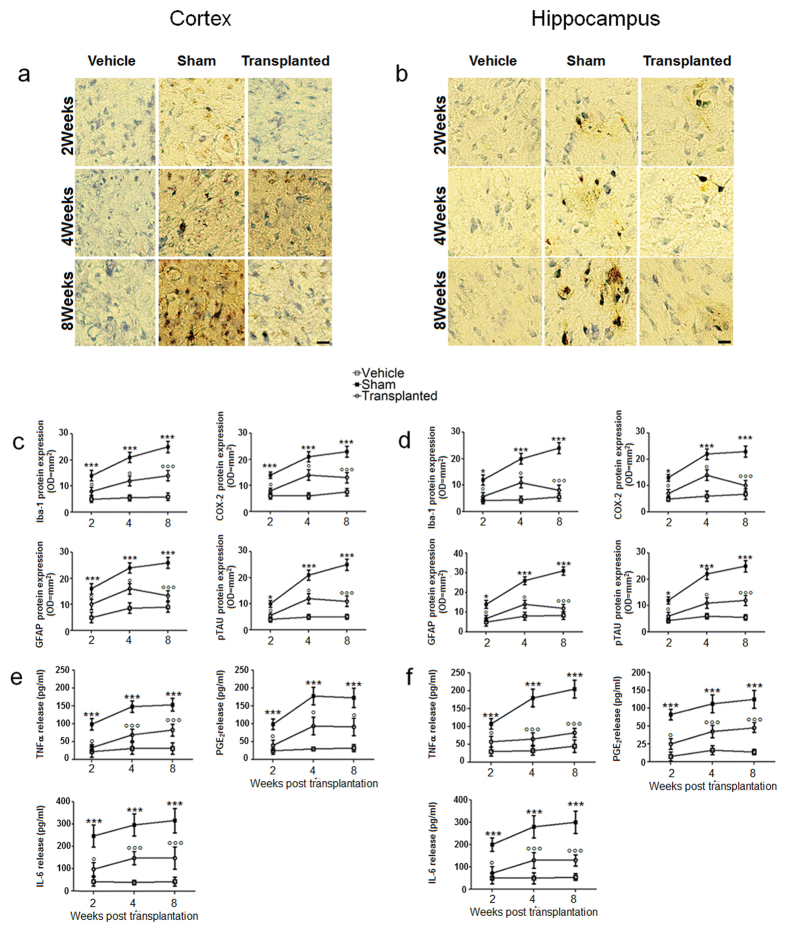
Autologous EGCs engraft reduces Aβ-dependent neuroinflammation. **(a,b)** Immunohistochemistry of phospho-tau (pSer202) in brain slices shows a significant and timely-course dependent increase in protein staining in sham (*n = *10) versus vehicle-treated rats (*n = *10) in frontal cortex (**a**) and hippocampus (**b**). Scale bar: 50 μm. **(c,d)** The graphs show the significant and time-dependent increased expression of Iba-1, COX-2, GFAP and phosphor-tau protein in the frontal cortex (**c**) and the hippocampus (**d**) of sham compared to vehicle-treated rats (*n = *10) (all P < 0.001). EGC transplantation caused a significant decrease in Iba-1, COX-2, GFAP and phosphor-tau protein expression along the time point intervals in both frontal cortex (**c**) and hippocampus (**d**) (°P < 0.05 and °°P < 0.001 vs. sham group). **(e,f)** The graphs show the level of pro-inflammatory cytokines measured by ELISA in the frontal cortex (**e**) and hippocampus (**f**) of sham, vehicle-treated and EGC-transplanted rats. Note the significant and time-dependent increase in TNF-α, PGE_2_ and IL-6 release (*P < 0.05 and ***P < 0.001) in sham compared to vehicle-treated rats. Autologous EGCs engraft caused a significant and time-dependent decrease in TNF-α, PGE_2_ and IL-6 release (*P < 0.05 and ***P < 0.001) in the frontal cortex (**e**) and the hippocampus (**f**) of EGC-transplanted versus sham rats. Results are expressed as mean ± S.E.M of *n = *5 experiments in triplicate for each animal group. Statistical analysis was performed using parametric one-way analysis of variance (ANOVA) and multiple comparisons were performed by Bonferroni’s post-test. Values of P < 0.05 were considered significant.

**Figure 4:**
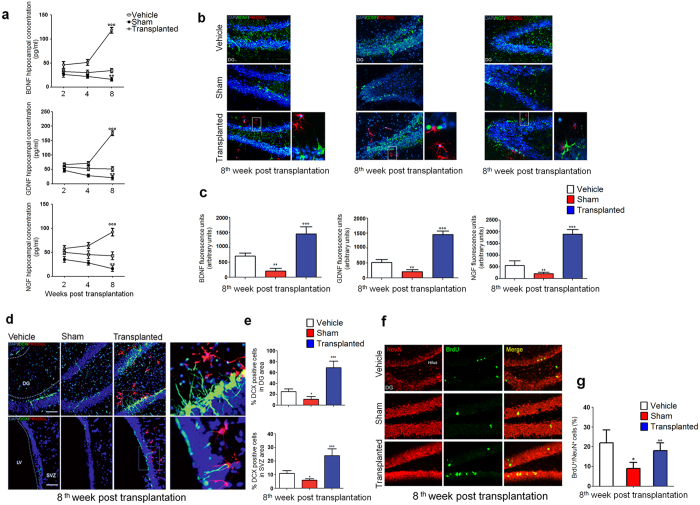
Autologous EGCs engraft increases neurotrophines release and neurogenesis. (**a**) Hippocampal release of NGF, BDNF and GDNF. A time-dependent decrease of neurotrophines was observed in sham compared to vehicle rats (*n = *8 and 6, respectively), with the lowest value at 8 weeks (**P < 0.01); in EGCs-transplanted rats the release of neurotrophines progressively increased, being maximum at 8 weeks (°°°*P* < 0.001; *n = *8). (**b**) Immunofluorescence of BDNF, GDNF and NGF (*all in green*) in dentate gyrus (DG) of isolated hippocampi; in EGCs-transplanted rats the close contact between PKH26GL-positive EGCs (*red*) and neurotrophines-expressing fibers (*green*) is magnified in the squares (*blue*: DAPI staining of nuclei; magnification: 10 and 25X; scale bar: 100 and 10μm). (**c**) Quantitative analysis of BDNF, GDNF and NGF showing that Aβ-associated reduction of neurotrophines fluorescence was significantly restored in EGC-transplanted rats (**P < 0.01 vs vehicle and °°°*P* < 0.001 vs. sham; *n = *6 per group). **(d)** Immunofluorescence of EGC (PKH26Gl, *red*), Doublecortin X (DCX, *green*) and DAPI (*blue*) in DG and subventricular zone (SVZ) showing the reduced neurogenesis in sham compared to vehicle rats (*n = *8, respectively). In EGC transplanted rats (*n = *8) the increased DCX immunopositivity was evident; a detail of the contact between EGCs and DCX arborizations is magnified in the square (magnification: 10 and 25X; Scale bar: 100 and 10 μm). **(e)** Quantitative analysis of positive DCX neurons in DG (*upper*) and SVZ (*lower*). The amount of DCX neurons was significantly decreased in sham rats, but in EGC-transplanted there was a significant increase in DCX percent (*P < 0.05 and °°°*P* < 0.001, respectively). Data are representative of mean ± S.E.M of n = 5 experiments in triplicate for each animal group; statistical analysis was performed using ANOVA and Bonferroni’s post-test; values of *P* < 0.05 were considered significant. (**f**) Immunofluorescence analysis of NeuN (*red)* and BrdU (*green*) in DG. At 8 weeks, BrdU positivity was reduced in sham rats, but it was increased in EGC-transplanted rats (magnification: 10X; scale bar: 100 μm). **(g)** Quantitative analysis showed that the percentage of NeuN/BrdU co-expressing neurons was significantly reduced in the sham group (*P < 0.05 vs. vehicle); in the transplanted group, NeuN/BrdU immunoreactivity was significantly increased (°°P < 0.01 vs sham), likely suggesting a remarkable rescue of neurogenesis.

**Figure 5:**
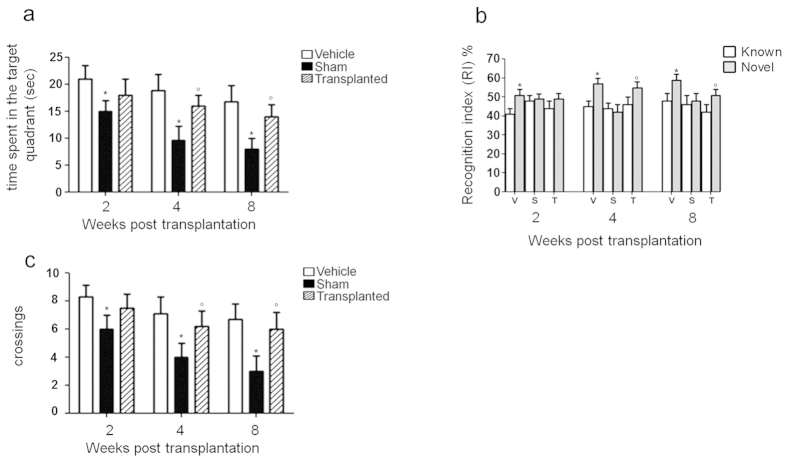
ECG transplantation improves Aβ-induced cognitive and behavior deficits. Morris water maze (MWM) tasks were performed on vehicle, sham and EGC transplanted animals at 2, 4 and 8 weeks post transplantation. **(a)** Time spent (measured in seconds) in the target quadrant (*P < 0.05 vs. vehicle; °P < 0.05 vs. sham; two-way ANOVA). **(b)** Number of crossings over the target quadrant was measured for 120 seconds at day 7 after the last acquisition test. MWM results shows that the sham group progressively decreased the time spent in the target quadrant and the number of crossings through the platform during the time course, compared to the vehicle group (*P < 0.05). EGC transplantation caused a progressive increase in the average time spent in the target quadrant resulting in a parallel increase in number of crossing on the platform; such increase began to be significant at week 4 and remained until week 8 post transplantation (°P < 0.05). Results presented in the graphs are representative of the mean ± S.E.M; *n = *10 animals per group. **(c)** To assess recognition memory, object recognition task was performed on vehicle, sham and transplanted animals at 2, 4 and 8 weeks post EGC transplantation. Recognition index (RI) defines the ratio of time spent exploring the novel object over the total time spent exploring both familiar and novel objects. In vehicle rats a significant increase in novel object RI was observed versus known object at all the time points (*P < 0.05). The same RI evaluation demonstrated non-significant variations in sham rats, whereas EGC transplantation almost completely restored the RI observed in vehicle rats (°P < 0.05). RI percent values were expressed for familiar and novel objects during the test task (after 3 h delay). Data are expressed as means ± SEM of *n = *10 experiments.
