# Supplementary material for: Autologous transplantation of intestine-isolated glia cells improves neuropathology and restores cognitive deficits in β amyloid-induced neurodegeneration
Source: Sci Rep. 2016 Mar 4;6:22605. doi: 10.1038/srep22605 (PMC4778118; doi:10.1038/srep22605)
Supplement: Supplementary Information [file srep22605-s1.doc]

**Supplementary Information**

**Autologous transplantation of intestine-isolated glia cells improves neuropathology and restores cognitive deficits in β amyloid-induced neurodegeneration**

Giuseppe Esposito^1*^, Giovanni Sarnelli^2*^, Elena Capoccia^1^, Carla Cirillo^3^, Marcella Pesce^2^, Jie Lu^4^, Gaetano Calì^5^, Rosario Cuomo^2^, and Luca Steardo^1^.

^1^Department of Physiology and Pharmacology, “La Sapienza” University of Rome, Italy

^2^Department of Clinical Medicine and Surgery, University of Naples "Federico II", Naples, Italy

^3^Laboratory for Enteric Neuroscience (LENS), TARGID, University of Leuven, Leuven, Belgium

^4^Department of Neurology, Beth Israel Deaconess Medical Center, Harvard Medical School, Boston, Massachusetts, USA

^5^Institute of Experimental Endocrinology and Oncology-CNR. Naples, Italy

**^*^** These authors contributed equally to this work

**Supplementary Figure 1**


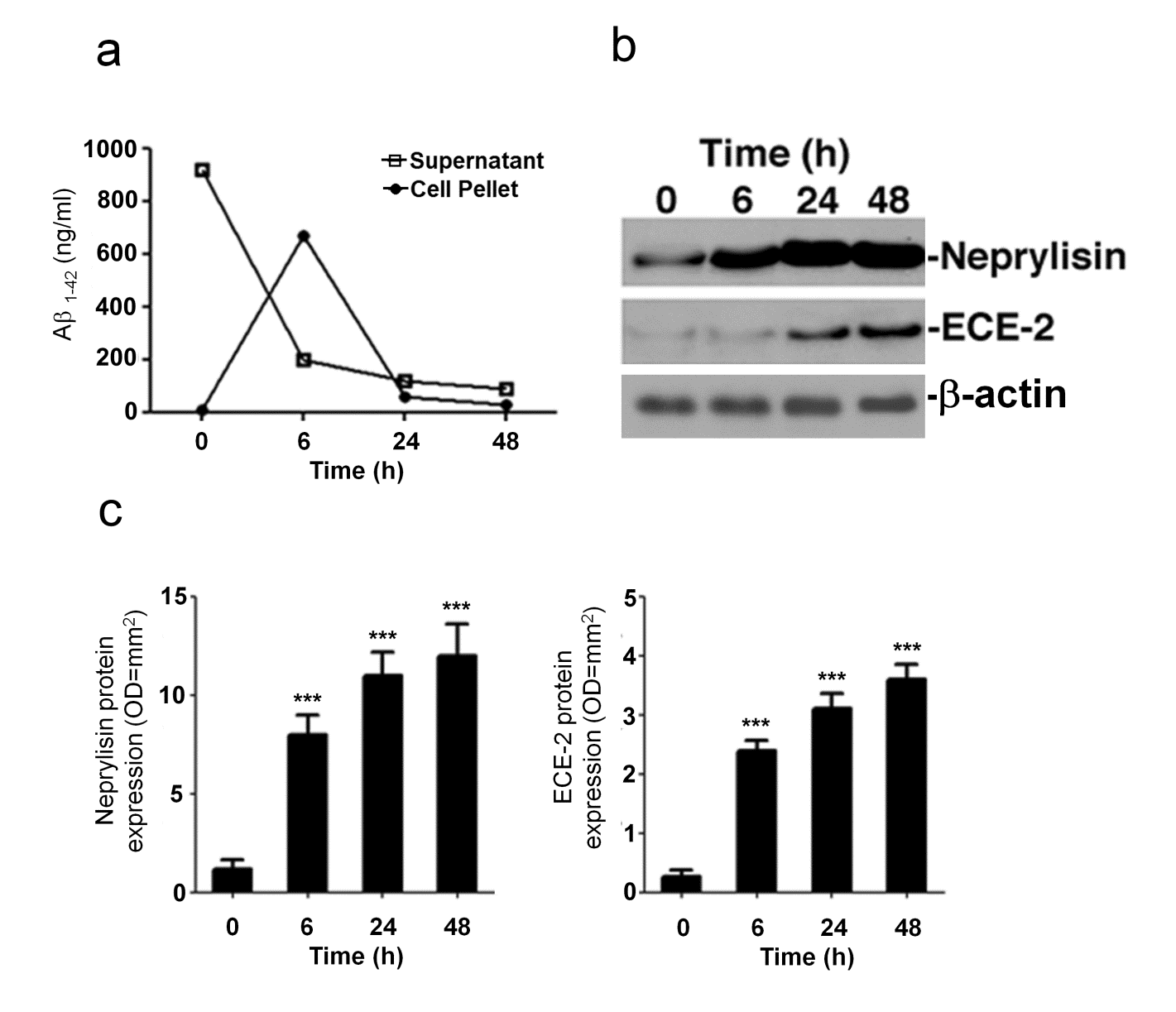


**Supplementary Figure 2**

**
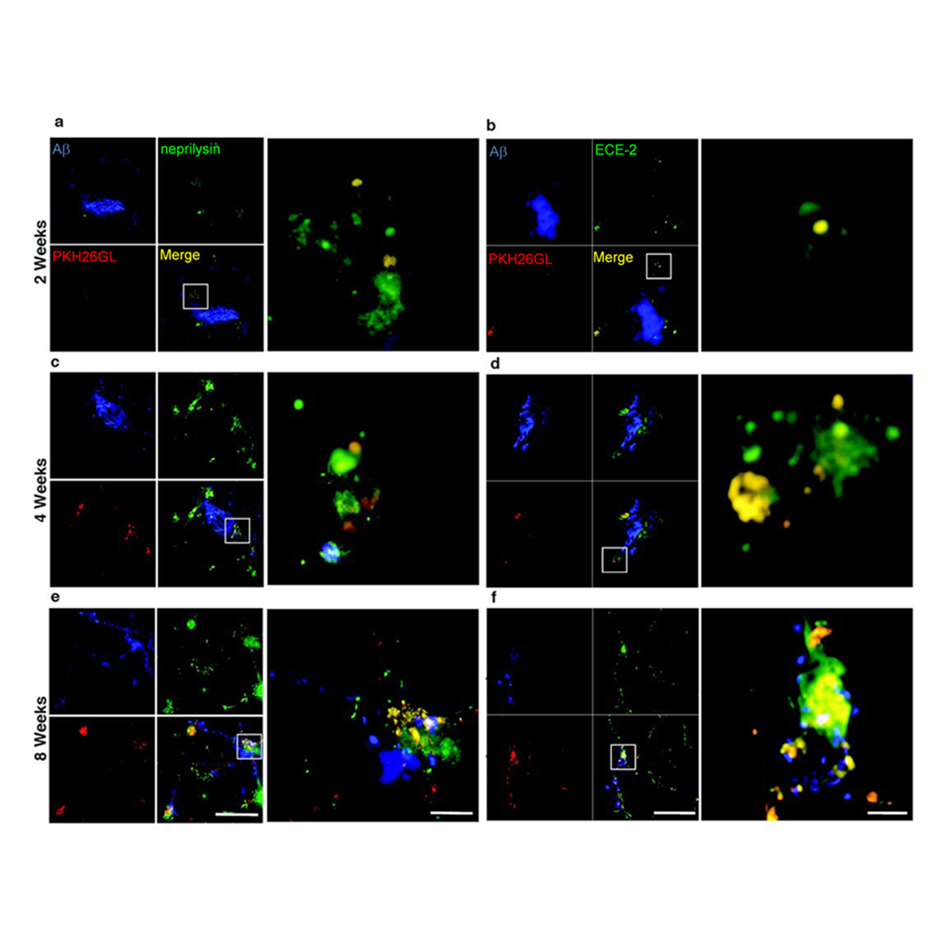
**

**Supplementary Figure 3**

**
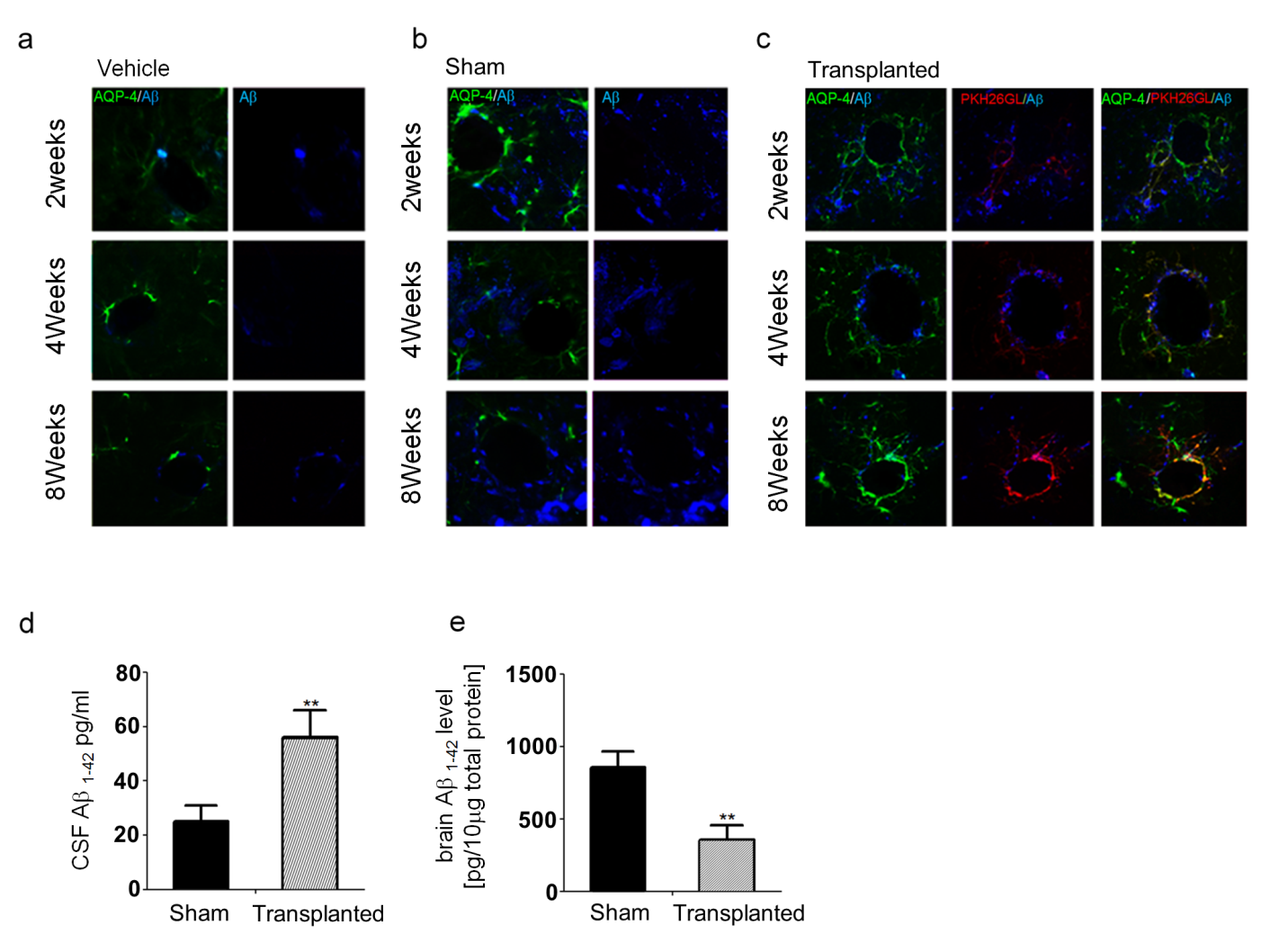
**

**Supplementary Figure 4**

**
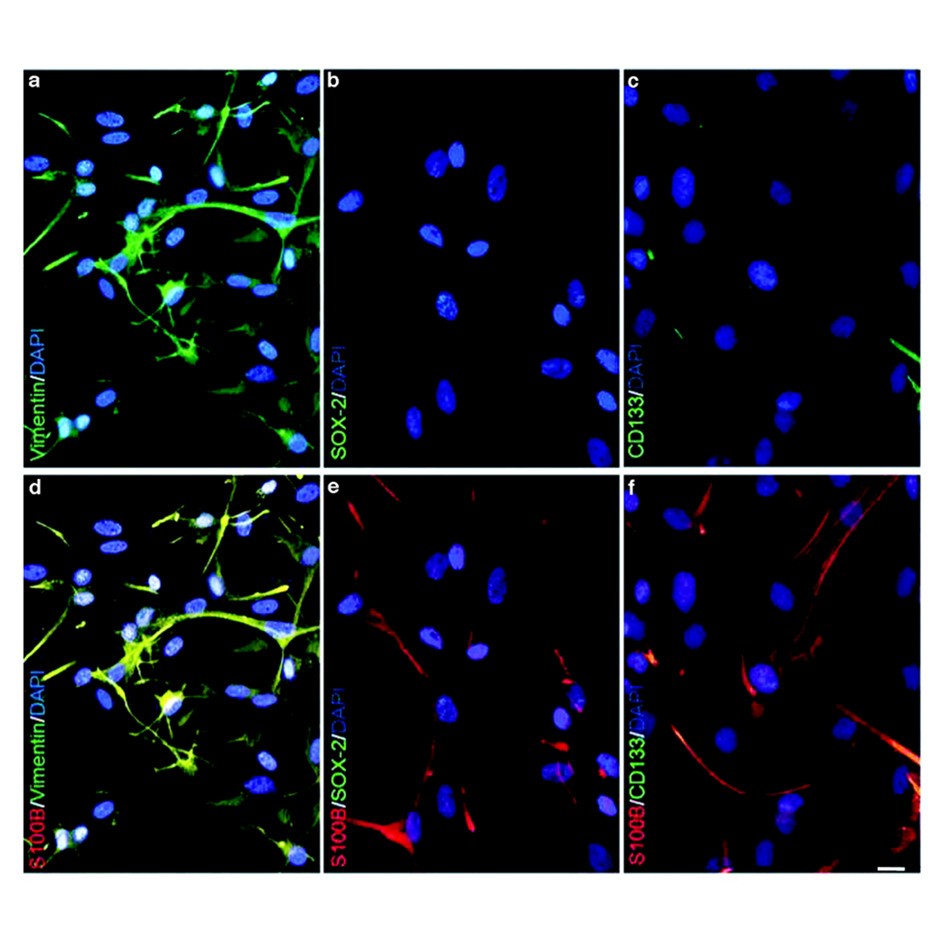
**

**Legends to supplementary figures**

**Figure 1. Cultured EGCs degrade Aβ and express neprilysin and ECE-2 *in vitro***

**(a)** The graph shows the level of Aβ_(1-42)_ *in vitro* in EGC cultures measured at 0, 6, 24 and 48h. Note the time-dependent decrease in Aβ_(1-42)_ in the supernatant of EGC culture (*squared line*) while in the pellet obtained after harvesting of EGCs the level of Aβ_(1-42)_ was significantly and time-dependently increased (*circled line*). **(b)** Representative Western Blot showing the protein expression of neprilysin and ECE-2 at 0, 6, 24 and 48h in EGC lysates. Note the strong and time-dependent increase in both proteins after addition of Aβ_(1-42)_ to EGC cultures. **(c)** Densitometric analysis of immunoblot bands for neprylisin and ECE-2 (*right* and *left panel,* respectively). Results are expressed as mean ± SEM of *n*=5 experiments performed in triplicate. ***P<0.001 vs 0 h time point. Statistical analysis was performed using parametric one-way analysis of variance (ANOVA) and multiple comparisons were performed by Bonferroni's post-test. Values of P<0.05 were considered significant.

**Figure 2. EGCs express neprilysin and ECE-2 enzyme, and degrade Aβ burdens in rat brains *in vivo*. (a, c, e)** Time course immunofluorescence analysis of EGCs (labeled with the tracer PKH26GL, *red*), neprilysin (*green*) and Aβ plaques (*blue*). **(b, d, f)** Time course immunofluorescence analysis of EGCs (PKH26GL, *red*), ECE-2 (*green*) and Aβ plaques (*blue*). Arrows indicate that transplanted EGCs (*red*) surround Aβ burdens (*blue*) and express both neprilysin (*green*) and ECE-2 (*green*) enzymes, demonstrating that EGCs are able to degrade Aβ in the rat brain. Pictures are representative of random frontal cortex or hippocampal areas analyzed in *n=*10 rat brains isolated at 2, 4 and 8 weeks post transplantation. Scale bar: 20 μm. The selected box shows that engrafted EGCs are capable to internalize Aβ. Scale bar: 10 μm.

**Figure 3. EGCs transplantation upregulates AQP-4 expression and contributes to Aβ clearance from tissue to cerebrospinal fluid**

Immunofluorescence showing transplanted EGCs (PKH26Gl, *red*), AQP-4 expression (*green*) and Aβ plaques (*blue*) in **(a)** vehicle-treated (*left*), **(b)** sham (*middle*) and **(c)** EGC-transplanted (*right*) rats at 2, 4 and 8 weeks (*top*, *middle* and *bottom* rows, respectively). Scale bar: 20 μm. A detectable amount of Aβ aggregates was present in the proximity of the vessels and a very low detection of AQP-4 protein was expressed in the brain of the sham group (*n=*6) (**b**, *middle*). Interestingly, in the transplanted group (*n=*6) AQP-4 expression (*green*) close to the vessels was significantly increased on engrafted EGCs (*red*) while a parallel decrease in Aβ immunoreactivity (*blue*) was observed (**c**). This effect was particularly evident at 8 weeks after EGC engrafts (**a**, *right*). **(d)** ELISA analyses showing that in EGC-transplanted rats a significantly higher concentration of Aβ was measured in CSF samples, than in sham rats (**P<0.01), and, accordingly **(e)** brain levels of Aβ were significantly lower (**P<0.01 vs. sham). Statistical analysis was performed using student-*t* test between two experimental groups; values of P<0.05 were considered significant.

**Figure 4. Isolated EGCs do not show stem cell-like profile**

Immunofluorescence showing the expression of vimentin alone (**a**, *green*) and merged with S100B (**b**, red). Note the matched signal (yellow) in the EGCs cultures. (**c**) Expression of SOX-2 (*green*) in S100B-positive (*red*) EGCs and merged image (**d**). (**e**) CD133 staining (*green*) in S100B-positive cultures and merged image (**f**). Note the absence of SOX-2 and CD133 in EGCs. Nuclei were stained with DAPI (*blue*). Scale bar: 10 µm.
